# Higher long-term visit-to-visit glycemic variability predicts new-onset atrial fibrillation in patients with diabetes mellitus

**DOI:** 10.1186/s12933-021-01341-3

**Published:** 2021-07-23

**Authors:** Jung-Chi Hsu, Yen-Yun Yang, Shu-Lin Chuang, Chih-Chieh Yu, Lian-Yu Lin

**Affiliations:** 1 Division of Cardiology, Department of Internal Medicine, Camillian Saint Mary’s Hospital Luodong, Yilan, Taiwan; 2 Division of Cardiology, Department of Internal Medicine, National Taiwan University College of Medicine and Hospital, No.7, Chung Shan South Road, 100 Taipei, Taiwan; 3 Graduate Institute of Epidemiology and Preventive Medicine, College of Public Health, National Taiwan University, Taipei, Taiwan; 4 Department of Medical Research, National Taiwan University Hospital, Taipei, Taiwan

**Keywords:** Glycemic variability, Atrial fibrillation, Diabetes mellitus

## Abstract

**Background:**

Atrial fibrillation (AF) is prevalent in patients with type 2 diabetes mellitus (T2DM). Glycemic variability (GV) is associated with risk of micro- and macrovascular diseases. However, whether the GV can increase the risk of AF remains unknown.

**Methods:**

The cohort study used a database from National Taiwan University Hospital, a tertiary medical center in Taiwan. Between 2014 and 2019, a total of 27,246 adult patients with T2DM were enrolled for analysis. Each individual was assessed to determine the coefficients of variability of fasting glucose (FGCV) and HbA1c variability score (HVS). The GV parameters were categorized into quartiles. Multivariate Cox regression models were employed to estimate the relationship between the GV parameters and the risk of AF, transient ischemic accident (TIA)/ischemic stroke and mortality in patients with T2DM.

**Results:**

The incidence rates of AF and TIA/ischemic stroke were 21.31 and 13.71 per 1000 person-year respectively. The medium follow-up period was 70.7 months. In Cox regression model with full adjustment, the highest quartile of FGCV was not associated with increased risk of AF [Hazard ratio (HR): 1.12, 95% confidence interval (CI) 0.96–1.29, p = 0.148] or TIA/ischemic stroke (HR: 1.04, 95% CI 0.83–1.31, p = 0.736), but was associated with increased risk of total mortality (HR: 1.33, 95% CI 1.12–1.58, p < 0.001) and non-cardiac mortality (HR: 1.41, 95% CI 1.15–1.71, p < 0.001). The highest HVS was significantly associated with increased risk of AF (HR: 1.29, 95% CI 1.12–1.50, p < 0.001), total mortality (HR: 2.43, 95% CI 2.03–2.90, p < 0.001), cardiac mortality (HR: 1.50, 95% CI 1.06–2.14, p = 0.024) and non-cardiac mortality (HR: 2.80, 95% CI 2.28–3.44, p < 0.001) but was not associated with TIA/ischemic stroke (HR: 0.98, 95% CI 0.78–1.23, p = 0.846). The Kaplan–Meier analysis showed significantly higher risk of AF, cardiac and non-cardiac mortality according to the magnitude of GV (log-rank test, p < 0.001).

**Conclusions:**

Our data demonstrate that high GV is independently associated with the development of new-onset AF in patients with T2DM. The benefit of maintaining stable glycemic levels to improve clinical outcomes warrants further studies.

**Supplementary Information:**

The online version contains supplementary material available at 10.1186/s12933-021-01341-3.

## Introduction

Atrial fibrillation (AF) is prevalent in patients with aging, congestive heart failure (CHF), hypertension (HTN), and diabetes mellitus (DM). Patients with type 2 diabetes mellitus (T2DM) carry an overall 35% higher risk of AF compared to general population, and increased blood glucose has a dose–response relationship with the incidence of AF [1, 2]. However, whether DM itself is an independent risk for AF or diabetic environment prone to AF has been controversial. Potential proarrhythmic mechanisms linking AF to DM are complex. Interstitial fibrosis and atrial dilatation, marker of DM atriopathy can cause structural remodeling. Decreased sodium ion channel, intracellular calcium dysregulation, and abnormal expression of connexins from connexin-40 to connexin-43, can change atrial refractoriness, resulting in electrical remodeling. Autonomic imbalance, sympathetic overactivation, and heterogeneous distribution of sympathetic nerves lead to autonomic remodeling [3, 4].

Apart from focusing primarily on measurement of fasting plasma glucose (FPG), hemoglobin A1c (HbA1c), advanced glycation end products (AGEs), glucagon-like peptide 1 (GLP-1), short-term glycemic variability within-days or months or even years have been considered as important risk factors for cardiovascular disease [5]. Glycemic fluctuation has been shown to over-activate oxidative stress and inflammation system, aggravating greater vascular damage and cardiomyopathy than that in chronic stable hyperglycemia [5, 6]. Increased glycemic variation (GV) also has adverse effect on autonomic function and increases the thrombotic properties of the platelets, which may be associated with higher incidence of major adverse cardiovascular event (MACE) [7, 8].

Previous studies have shown that patients with higher acute GV have more vulnerable plaques and poorer prognosis of acute coronary syndrome [9–11]. Short-term GV is also associated with increased mortality after cardiac procedure such as transcatheter aortic valve implantation [12]. Long-term GV was found to be associated with greater progression of coronary artery calcification in young adults [13]. High GV also causes left ventricular diastolic dysfunction and increased the risk of heart failure [14, 15]. In the Antihypertensive and Lipid-Lowering Treatment to Prevent Heart Attack Trial (ALLHAT) study, GV was independently associated with the risks of cardiovascular event and all-cause mortality [16].

Nevertheless, the majority of the studies regarding the effect of GV have focused on diabetic macro- and micro-vascular complications. Whether GV is associated with the development of AF is not known. Investigating the contribution of GV on the development of AF may advance our understanding of how dysfunction in glucose homeostasis impacts atrial remodeling. In this cohort study, we plan to investigate the association of long-term GV with the incidence of AF and related cardiovascular outcomes in a group of patients with T2DM.

## Methods

### Study population and data collection

The study was approved by the Institutional Review Board (IRB) of National Taiwan University Hospital. The study database was from National Taiwan University Hospital integrated Medical Database (NTUH-iMD) which was composed of detailed medical information from a tertiary medical center in Taiwan. Since AF is prevalent in patients with older age, we enrolled those with age above 50 years and diagnosed with T2DM at the National Taiwan University Hospital from January 1, 2014, to December 31, 2019. Patients with previous AF or lost to follow-up (defined as an absence of follow-up at the outpatient clinics between Oct. 1st to Dec. 31, 2019 since we followed patients at least every three months) were excluded. For simplicity, patients who had severe end organ damage including history of congestive heart failure (CHF) or above stage 3 chronic kidney disease (CKD) were also excluded. Baseline characteristics including body mass index (BMI), hypertension (HTN), hyperlipidemia, coronary artery disease (CAD), acute coronary syndrome (ACS), myocardial infarction (MI), chronic obstructive pulmonary disease (COPD), peripheral arterial occlusive disease (PAOD) were obtained from the electronic health records (EHRs). Estimated glomerular filtration rate (eGFR) was calculated by modification of diet in renal disease (MDRD) equation. Prescription information were categorized into antiarrhythmic agents, calcium channel blocker (CCB), beta-blocker, angiotensin converting enzyme inhibitor (ACEI), angiotensin receptor blocker (ARB), mineralocorticoid-receptor antagonist (MRA), anticoagulants including direct oral anticoagulant (DOAC) and warfarin, and anti-diabetic medications including insulin, metformin, sodium–glucose co-transporter-2 (SGLT2) inhibitor, dipeptidyl peptidase 4 (DPP4) inhibitor, sulphonylurea, repaglinide, acarbose, thiazolidinedione (TZD), glucagon like peptide-1 (GLP-1) agonist. Echocardiographic studies were performed with Phillips iE33 (Phillips, Bothell, WA, USA) and two‐dimensional‐guided M‐mode measurements with a 3.0‐ or 3.5‐MHz transducer. Left atrium (LA) size, LV internal dimension in end‐diastole (LVIDd) and systole (LVIDs), and LV ejection function were collected in the parasternal long-axis view with M-mode cursor. LA size was anterior–posterior diameter measured at the end-ventricular systolic phase. LV mass was calculated by using the Devereux formula. All the echocardiographic data were assessed from the EHRs.

The outcomes were AF, transient ischemic accident (TIA), ischemic stroke and mortality. Death events were also adjudicated by a central committee, and were separated to cardiac and noncardiac mortality. AF and its occurrence time were identified by the diagnosis code from either the EHRs or the standard 12-lead electrocardiogram. The index dates of outcomes were defined as the dates of diagnosis. All medical records were reviewed until their last clinical visit or death.

### Glycemic variability measurement

We calculated two measures of GV for each individual: the coefficient variability of the mean FPG (FGCV) and the HbA1c variability score (HVS). FPG and HA1C were measured quarterly at visit-to-visit outpatient department. FPG was measured in subjects who reported fasting more than 8 h. The calculation of FPG and HbA1c variability was from at least three successive measurements. Only patients having both FGCV and HVS were included for analysis. FGCV (%) was calculated as the standard deviation (SD) of FPG divided by the mean FPG, and then divided by the square root of the ratio of FPG measurements n to n − 1 ($\sqrt {n/(n - 1)}$) to account for the influence of FPG measurement number [17, 18]. HVS is the number of measures within an individual where the HbA1c has changed by > 0.5% (5.5 mmol/mol) from the prior value, as a percentage of the total number of HbA1c measures between the diagnosis of diabetes and the outcome. In brief, HVS is a percentage of HbA1c fluctuation events (Δ > 0.5%) [19].

### Statistical analysis

Patients were categorized into four groups according to the quartiles of FGCV or HVS. Continuous variables were described as mean (SD) and categorical variables were reported as frequency (percentage). Differences among groups were tested by using chi square test for categorical variables and analysis of variance (ANOVA) test for continuous variables. Multivariate Cox proportional hazards models were constructed to assess the association of categorical and continuous measures. The relationship between GV and the development of diabetes complications was assessed by Cox regression from which hazard ratios (HRs) and 95% confidence intervals (CIs) were derived. The assumption of proportionality was validated by verifying the Schoenfeld residuals. Trend analysis in the general linear model and Cochrane-Armitage test were used for evaluating dose–response effects.

The semiparametric Cox regression models were sequentially adjusted for the following baseline covariates. The model 1 was crude model without any adjustment. The model 2 adjusted for age, gender (male as reference group), baseline BMI, history of HTN, COPD, CAD, PAOD and prior TIA/ischemic stroke, baseline FPG, baseline HbA1c, and baseline estimated glomerular filtration rate (eGFR). The model 3 adjusted for model 2 plus three echocardiogram parameters including LA size, LVEF, LVM. Model 4 adjusted for model 3 plus the medications including antiplatelet, anticoagulant, CCB, betablocker, ACEI/ARB, diuretic, statin, insulin, metformin, SGLT2 inhibitor, DDP4 inhibitor, sulphonylurea, repaglinide, acarbose, TZD, GLP-1 agonist. Survival analyses were presented by using Kaplan–Meier curves and the significance of difference between curves were examined by log-rank test. All statistical analyses were performed using SAS statistical software package (version 9.4. SAS Institute Inc., Cary, NC, USA). A two-tailed p-value of less than 0.05 was considered statistically significant.

## Results

### Baseline characteristics

**Fig. 1:**
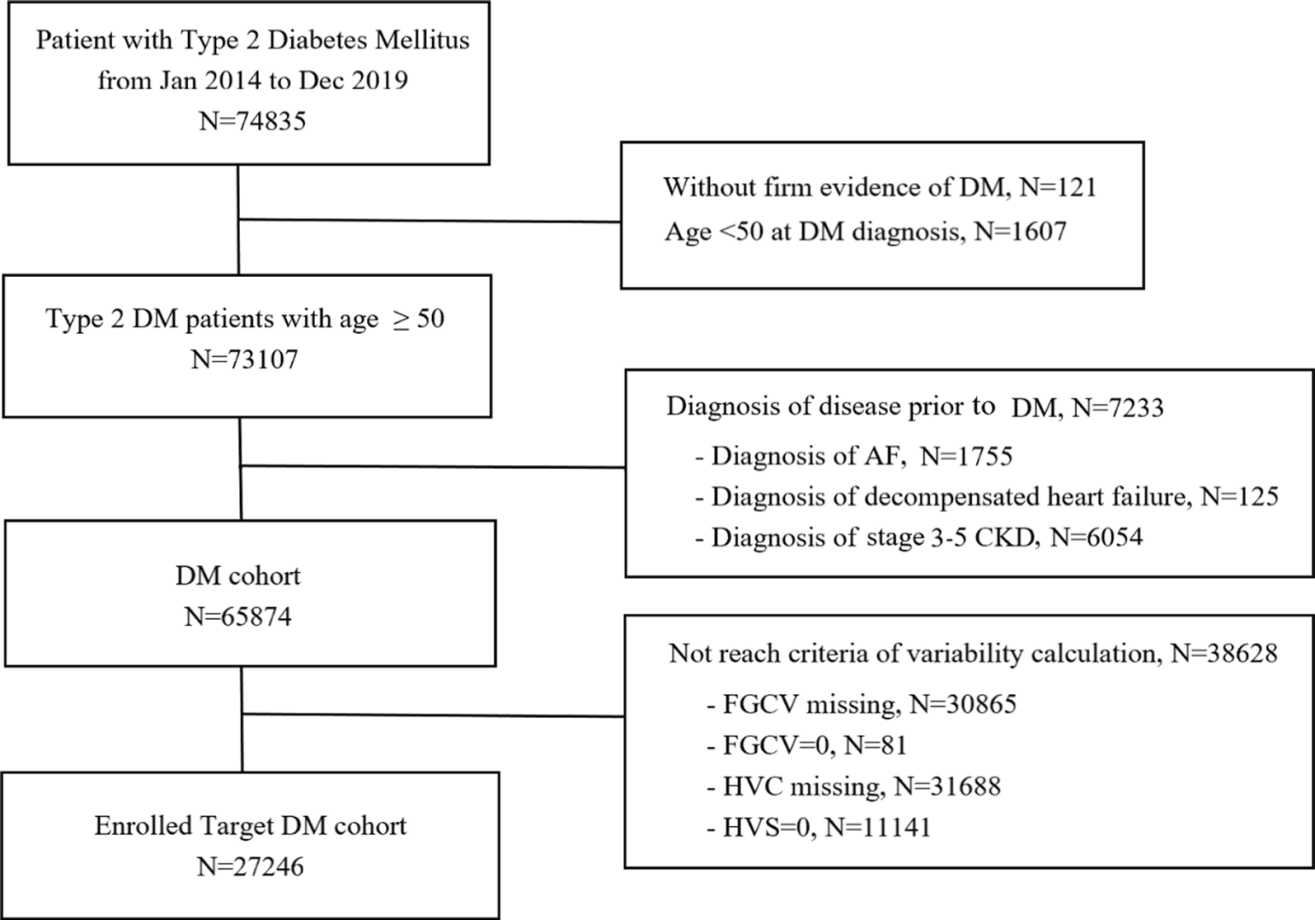
Flow chart of patient selection

The flowchart of patient selection was demonstrated in Fig. 1. A total of 74,835 with T2DM diagnosis code between 2014 and 2019 were enrolled. Among them, 121 patients without firm evidence of T2DM (blood test, DM medications) and 1607 patients aged below 50 years were excluded. We excluded 1755 patients with pre-existing AF, 125 patients with severe CHF (ever hospitalization caused by acute decompensated heart failure), and 6054 patients with moderate or severe CKD (≥ stage 3 CKD). We only included patients with both FGCV and HVS data, so 38,628 patients who only had either FGCV or HVS data were excluded. We also excluded the patients with FGCV = 0 (n = 81) or HVS = 0 (n = 11,141, over 70% < 10 measurements) to simplify the analysis and uniform the approach for FGCV and HVS. Finally, a total of 27,246 subjects were enrolled for further analysis. The patients were grouped according to the quartiles of either FGCV or HVS. The clinical, biochemical and anthropometric characteristics were presented in Table 1. The subjects in the highest quartile of FGCV were older, had higher baseline FPG and HbA1c, had worse baseline eGFR, were more likely to have PAOD and less likely to have HTN or CAD. The subjects in the highest quartile of HVS were more male gender, had less HTN, had higher baseline FPG and HbA1c levels and lower baseline eGFR.

**Table 1:** Baseline patients’ characteristics (N = 27,246)

	FGCV				p value	HVS				p value
	Q1 (0.34–11.69)	Q2 (11.70–17.59)	Q3 (17.60–26.19)	Q4 (26.20–188.12)		Q1 (1.37–18.17)	Q2 (18.18–33.99)	Q3 (34.00–51.10)	Q4 (51.11–100.00)	
N	6726 (24.69)	7099 (26.06)	6641 (24.37)	6780 (24.88)		6778 (24.88)	7855 (28.83)	6396 (23.48)	6217 (22.82)	
Age (year)	66.49 (9.38)	66.45 (9.51)	66.67 (9.75)	67.08 (10.14)	< 0.001	66.59 (9.27)	66.76 (9.63)	66.73 (9.95)	66.58 (9.97)	0.598
Male	3576 (53.17)	3808 (53.64)	3446 (51.89)	3672 (54.16)	0.054	3457 (51.00)	4167 (53.05)	3432 (53.66)	3446 (55.43)	< 0.001
Baseline BMI (kg/m^2^)	25.70 (4.00)	25.82 (3.94)	25.87 (4.47)	25.52 (4.37)	< 0.001	25.57 (3.90)	25.78 (4.04)	25.92 (4.57)	25.63 (4.29)	< 0.001
Hypertension	1188 (17.66)	1014 (14.28)	795 (11.97)	689 (10.16)	< 0.001	1114 (16.44)	1129 (14.37)	779 (12.18)	664 (10.68)	< 0.001
COPD	102 (1.52)	83 (1.17)	70 (1.05)	88 (1.30)	0.094	85 (1.25)	101 (1.29)	81 (1.27)	76 (1.22)	0.990
CAD	498 (7.40)	439 (6.18)	396 (5.96)	352 (5.19)	< 0.001	413 (6.09)	506 (6.44)	382 (5.97)	384 (6.18)	0.685
PAOD	51 (0.76)	41 (0.58)	61 (0.92)	72 (1.06)	0.011	47 (0.69)	74 (0.94)	57 (0.89)	47 (0.76)	0.327
History of TIA/stroke	52 (0.77)	52 (0.73)	68 (1.02)	62 (0.91)	0.232	47 (0.69)	62 (0.79)	71 (1.11)	54 (0.87)	0.061
Baseline FPG (mg/dL)	127.75 (25.14)	136.41 (32.37)	146.04 (45.97)	165.60 (86.89)	< 0.001	128.06 (30.78)	138.59 (43.06)	149.87 (61.03)	161.69 (73.27)	< 0.001
Baseline HbA1c (%)	6.94 (1.06)	7.22 (1.16)	7.64 (1.46)	8.21 (1.83)	< 0.001	6.83 (0.95)	7.30 (1.26)	7.71 (1.47)	8.24 (1.82)	< 0.001
Baseline eGFR (mL/min/1.73 m^2^)	77.85 (28.76)	75.73 (28.92)	72.52 (31.89)	64.96 (34.48)	< 0.001	74.42 (28.04)	73.54 (30.63)	71.44 (32.36)	71.36 (34.87)	< 0.001
CHA2DS2-VASc score	2.49 (1.10)	2.44 (1.09)	2.46 (1.12)	2.45 (1.13)	0.042	2.49 (1.09)	2.48 (1.11)	2.45 (1.14)	2.40 (1.11)	< 0.001
Echocardiography										
LA size (cm)	3.89 (0.67)	3.91 (0.65)	3.90 (0.65)	3.93 (0.68)	0.139	3.91 (0.63)	3.92 (0.66)	3.91 (0.66)	3.90 (0.69)	0.733
DT (s)	0.22(0.07)	0.23 (0.06)	0.23 (0.06)	0.22 (0.07)	< 0.001	0.23 (0.06)	0.22 (0.06)	0.22 (0.07)	0.22 (0.07)	< 0.001
E (mm/s)	77.62 (24.73)	78.97(25.02)	80.50 (26.83)	84.70 (29.17)	< 0.001	78.69 (24.39)	80.51 (26.24)	81.22 (27.69)	82.42 (28.55)	< 0.001
A (mm/s)	95.13 (22.88)	97.18 (23.29)	98.73 (24.30)	100.54 (26.25)	< 0.001	97.40 (22.60)	97.64 (24.94)	98.36 (24.13)	98.93 (25.69)	0.174
E/A	0.84 (0.39)	0.91 (3.30)	0.84 (0.49)	0.94 (3.12)	0.360	0.82 (0.37)	0.85 (0.56)	0.98 (4.52)	0.88 (1.04)	0.157
E/Eʹ	26.72 (173.99)	17.17 (68.09)	18.21 (80.74)	22.49 (120.79)	0.675	30.50 (175.67)	15.26 (52.97)	24.00 (129.85)	19.58 (109.46)	0.369
LVEF (%)	66.21 (11.09)	65.72 (11.48)	64.97 (11.84)	63.02 (13.09)	< 0.001	66.97 (10.74)	65.39 (11.67)	63.99 (12.49)	63.21 (12.49)	< 0.001
LVIDs (cm)	2.99 (0.66)	3.01 (0.67)	3.03 (0.66)	3.09 (0.74)	< 0.001	2.96 (0.62)	3.03 (0.67)	3.07 (0.71)	3.08 (0.73)	< 0.001
LVIDd (cm)	4.76 (0.60)	4.78 (0.59)	4.78 (0.59)	4.77 (0.65)	0.825	4.78 (0.56)	4.78 (0.60)	4.78 (0.62)	4.75 (0.65)	0.304
LV mass (gm)	199.69 (59.25)	201.15(59.57)	204.25 (57.80)	208.61 (64.07)	< 0.001	200.24 (57.41)	201.81 (57.87)	206.87 (62.90)	206.12 (63.46)	< 0.001
TR Vmax (cm/s)	252.82 (46.28)	252.82(46.37)	252.85 (48.64)	257.39 (52.05)	< 0.001	255.17 (44.75)	253.30 (47.15)	253.27 (49.34)	255.24 (52.92)	0.317
Medication										
Antiplatelet	2706 (40.23)	3098 (43.64)	3028 (45.60)	3236 (47.73)	< 0.001	2724 (40.19)	3457 (44.01)	3022 (47.25)	2865 (46.08)	< 0.001
Anticoagulant	351 (5.22)	359 (5.06)	390 (5.87)	471 (6.95)	< 0.001	339 (5.00)	447 (5.69)	396 (6.19)	389 (6.26)	0.006
CCB	3510 (52.19)	3937 (55.46)	3856 (58.06)	4153 (61.25)	< 0.001	3699 (54.57)	4435 (56.46)	3774 (59.01)	3548 (57.07)	< 0.001
Beta-blocker	2541 (37.78)	2760 (38.88)	2697 (40.61)	2983 (44.00)	< 0.001	2610 (38.51)	3154 (40.15)	2678 (41.87)	2539 (40.84)	0.001
ACEI/ARB	3956 (58.82)	4528 (63.78)	4364 (65.71)	4466 (65.87)	< 0.001	4280 (63.15)	5049 (64.28)	4144 (64.79)	3841 (61.78)	0.002
Diuretics	1673 (24.87)	1975 (27.82)	2246 (33.82)	3078 (45.40)	< 0.001	1751 (25.83)	2451 (31.20)	2356 (36.84)	2414 (38.83)	< 0.001
Statin	3820 (56.79)	4210 (59.30)	3995 (60.16)	3888 (57.35)	< 0.001	4096 (60.43)	4676 (59.53)	3777 (59.05)	3364 (54.11)	< 0.001
Propafenone	165 (2.45)	171 (2.41)	163 (2.45)	146 (2.15)	0.609	186 (2.74)	199 (2.53)	145 (2.27)	115 (1.85)	0.006
Amiodarone	327 (4.86)	386 (5.44)	463 (6.97)	704 (10.38)	< 0.001	348 (5.13)	494 (6.29)	490 (7.66)	548 (8.81)	< 0.001
Insulin	946 (14.06)	1446 (20.37)	2458 (37.01)	4257 (62.79)	< 0.001	1049 (15.48)	2263 (28.81)	2700 (42.21)	3095 (49.78)	< 0.001
Metformin	5406 (80.37)	6081 (85.66)	5532 (83.30)	4994 (73.66)	< 0.001	5698 (84.07)	6477 (82.46)	5134 (80.27)	4704 (75.66)	< 0.001
SGLT-2 inhibitor	808 (12.01)	1370 (19.30)	1461 (22.00)	1147 (16.92)	< 0.001	858 (12.66)	1450 (18.46)	1326 (20.73)	1152 (18.53)	< 0.001
DPP4 inhibitor	2908 (43.24)	4225 (59.52)	4437 (66.81)	4758 (70.18)	< 0.001	3172 (46.80)	4710 (59.96)	4310 (67.39)	4136 (66.53)	< 0.001
Sulphonylurea	2395 (35.61)	4416 (62.21)	4824 (72.64)	4684 (69.09)	< 0.001	3174 (46.83)	4779 (60.84)	4236(66.23)	4130 (66.43)	< 0.001
Novonorm	354 (5.26)	664 (9.35)	876 (13.19)	1307 (19.28)	< 0.001	513 (7.57)	857 (10.91)	867 (13.56)	964 (15.51)	< 0.001
Acarbose	703 (10.45)	1119 (15.76)	1378 (20.75)	1678 (24.75)	< 0.001	863 (12.73)	1375 (17.50)	1367 (21.37)	1273 (20.48)	< 0.001
TZD	619 (9.20)	1303 (18.35)	1670 (25.15)	1722 (25.40)	< 0.001	992 (14.64)	1494 (19.02)	1433 (22.40)	1395 (22.44)	< 0.001
GLP-1 agonist	37 (0.55)	132 (1.86)	261(3.93)	331 (4.88)	< 0.001	90 (1.33)	175 (2.23)	252 (3.94)	244 (3.92)	< 0.001

*FGCV* coefficients of variability of fasting glucose, *HVS* HbA1c variability score, *BMI* body mass index, *COPD* chronic obstructive pulmonary disease, *CAD* coronary artery disease, *PAOD* peripheral arterial occlusive disease, *FPG* fasting glucose, *eGFR* estimated glomerular filtration rate, *LA* left atrium, *DT* deceleration time, *E/A* early diastolic transmitral flow velocity/late diastolic transmitral flow velocity, *E*ʹ early diastolic mitral annular velocity, *LVEF* left ventricular ejection fraction, *LVIDd* left ventricular internal diameter in diastole, *LVIDs* left ventricular internal diameter in systole, *LV mass* left ventricle mass, *TR Vmax* maximal tricuspid regurgitation velocity, *CCB* calcium channel blocker, *ACEI/ARB* angiotensin converting enzyme inhibitor/angiotensin receptor blocker, *SGLT-2 inhibitor* sodium–glucose co-transporter-2 inhibitor, *DPP4 inhibitor* dipeptidyl peptidase 4 inhibitor, *TZD* thiazolidinediones, *GLP-1 agonist* glucagon like peptide-1 agonist

Over a median follow-up of 70.7 months, there were 2762 AF events (overall incidence rate 21.31 per 1000 person-year) and 1803 TIA/ischemic stroke events (overall incidence rate 13.71 per 1000 person-year). The incidence rates of AF for FGCV quartiles were 16.47, 17.66, 19.86, and 31.76 per 1000 person-year. The incidence rates of AF for HVS quartiles were 14.19, 19.30, 24.37, and 29.27 per 1000 person-year. The incidence rates of TIA/ischemic stroke for FGCV quartiles were 11.98, 11.52, 13.88, 17.66 per 1000 person-year. The incidence rates of TIA/ischemic stroke for HVS quartiles were 10.32, 15.83, 16.52, 15.63 per 1000 person-year. There were 3545 deaths in which 615 (17.35%) were cardiac causes.

### Measures of glycemic variability and outcomes

As shown in Table 2, in comparison with the first quartile of FGCV, the hazard ratios (HRs) across quartiles (second to fourth quartiles) for AF were 1.09 (95% CI 0.97–1.23, p = 0.137), 1.24 (95% CI 1.10–1.39, p < 0.001), and 2.00 (95% CI 1.80–2.22, p < 0.001) in model 1 without adjustment. After model 2 and 3 adjustment, the fourth quartile remained significantly associated with the development of AF and the HRs were 1.74 (95% CI 1.55–1.96, p < 0.001) and 1.45 (95% CI 1.26–1.66, p < 0.001) for model 2 and 3 respectively. In fully adjusted model 4, the HRs for AF became insignificant (1.12, 95% CI 0.96–1.29, p = 0.148). As for other outcomes, in fully adjusted model, the fourth quartile remained significant for total mortality (1.33, 95% CI 1.12–1.58, p < 0.001) and non-cardiac mortality (1.41, 95% CI 1.15–1.71, p < 0.001) but became insignificant for TIA/ischemic stroke (1.04, 95% CI 0.83–1.31, p = 0.736) and cardiac mortality (1.05, 95% CI 0.72–1.52, p = 0.810).

**Table 2:** Adjusted hazard ratios for AF, TIA/ischemic stroke, cardiac mortality and total mortality across quartiles of glycemic variability

Outcome	Group	No	Event (%)	Model 1		Model 2		Model 3		Model 4	
				HR (95% C.I.)	p	HR (95% C.I.)	p	HR (95% C.I.)	p	HR (95% C.I.)	p
AF	FGCV_Q1	6726	514 (7.64)	ref		ref		ref		ref	
	FGCV_Q2	7099	616 (8.68)	1.09 (0.97–1.23)	0.137	1.08 (0.96–1.22)	0.199	1.11 (0.97–1.28)	0.138	1.09 (0.94- 1.25)	0.259
	FGCV_Q3	6641	643 (9.68)	1.24 (1.10–1.39)	< 0.001	1.18 (1.04–1.33)	0.008	1.12 (0.97–1.28)	0.128	0.99 (0.85- 1.14)	0.850
	FGCV_Q4	6780	989 (14.59)	2.00 (1.80–2.22)	< 0.001	1.74 (1.55–1.96)	< 0.001	1.45 (1.26–1.66)	< 0.001	1.12 (0.96- 1.29)	0.148
	P for trend			< 0.001				< 0.001		0.2839	
TIA / stroke	FGCV_Q1	6726	376 (5.59)	ref		ref		ref		ref	
	FGCV_Q2	7099	407 (5.73)	1.00 (0.87–1.15)	0.971	0.98 (0.85–1.14)	0.823	1.10 (0.88–1.36)	0.413	0.98 (0.78- 1.23)	0.860
	FGCV_Q3	6641	453 (6.82)	1.21 (1.05–1.38)	0.007	1.06 (0.92–1.23)	0.402	1.13 (0.91–1.40)	0.270	0.92 (0.73–1.16)	0.497
	FGCV_Q4	6780	567 (8.36)	1.53 (1.34–1.74)	< 0.001	1.34 (1.16–1.55)	< 0.001	1.34 (1.08–1.66)	0.007	1.04 (0.83–1.31)	0.736
	P for trend			< 0.001		< 0.001		0.006		0.785	
Total mortality	FGCV_Q1	6726	522 (7.76)	ref		ref		ref		ref	
	FGCV_Q2	7099	615 (8.66)	1.03 (0.92–1.16)	0.592	1.03 (0.91–1.16)	0.649	1.05 (0.88–1.27)	0.565	0.96 (0.79–1.15)	0.639
	FGCV_Q3	6641	816 (12.29)	1.50 (1.34–1.67)	< 0.001	1.43 (1.27–1.61)	< 0.001	1.31 (1.10–1.56)	0.003	1.00 (0.84–1.20)	0.967
	FGCV_Q4	6780	1589 (23.44)	3.15 (2.85–3.48)	< 0.001	2.70 (2.42–3.02)	< 0.001	2.38 (2.02–2.80)	< 0.001	1.33 (1.12–1.58)	0.001
	P for trend			< 0.001		< 0.001		< 0.001		< 0.001	
Cardiac mortality	FGCV_Q1	6726	92 (1.37)	ref		ref		ref		ref	
	FGCV_Q2	7099	111 (1.56)	1.05 (0.80–1.39)	0.714	1.02 (0.76–1.37)	0.901	1.00 (0.68–1.46)	0.992	0.97 (0.66–1.42)	0.869
	FGCV_Q3	6641	142 (2.14)	1.47 (1.13–1.91)	0.003	1.18 (0.89–1.58)	0.256	1.07 (0.74–1.55)	0.723	0.90 (0.61–1.33)	0.609
	FGCV_Q4	6780	270 (3.98)	3.04 (2.40–3.85)	< 0.001	1.91 (1.45–2.51)	< 0.001	1.53 (1.08–2.17)	0.017	1.05 (0.72–1.52)	0.810
	P for trend			< 0.001		< 0.001		< 0.001		0.5772	
Non-cardiac mortality	FGCV_Q1	6726	430 (6.39)	ref		ref		ref		ref	
	FGCV_Q2	7099	504 (7.10)	1.03 (0.90–1.17)	0.675	1.03 (0.90–1.18)	0.678	1.07 (0.87–1.32)	0.532	0.95 (0.77–1.18)	0.647
	FGCV_Q3	6641	674 (10.15)	1.50 (1.33–1.69)	< 0.001	1.48 (1.30–1.68)	< 0.001	1.38 (1.13–1.68)	0.002	1.03 (0.84–1.27)	0.770
	FGCV_Q4	6780	1319 (19.45)	3.17 (2.84–3.54)	< 0.001	2.86 (2.53–3.23)	< 0.001	2.67 (2.22–3.21)	< 0.001	1.41 (1.15–1.71)	< 0.001
	P for trend			< 0.001		< 0.001		< 0.001		< 0.001	
AF	HVS_Q1	6778	518 (7.64)	ref		ref		ref		ref	
	HVS_Q2	7855	733 (9.33)	1.30 (1.16–1.45)	< 0.001	1.29 (1.15–1.45)	< 0.001	1.22 (1.07–1.40)	0.003	1.11 (0.97–1.27)	0.138
	HVS_Q3	6396	732 (11.44)	1.64 (1.47–1.84)	< 0.001	1.60 (1.42–1.80)	< 0.001	1.37 (1.20–1.57)	< 0.001	1.17 (1.02–1.34)	0.024
	HVS_Q4	6217	779 (12.53)	1.97 (1.76–2.20)	< 0.001	1.99 (1.77–2.25)	< 0.001	1.57 (1.37–1.81)	< 0.001	1.29 (1.12–1.50)	< 0.001
	P for trend			< 0.001		< 0.001		< 0.001		< 0.001	
TIA / stroke	HVS_Q1	6778	363 (5.36)	ref		ref		ref		ref	
	HVS_Q2	7855	507 (6.45)	1.25 (1.09–1.43)	0.001	1.22 (1.06–1.40)	0.006	1.22 (0.99–1.50)	0.059	1.07 (0.87–1.32)	0.515
	HVS_Q3	6396	504 (7.88)	1.55 (1.35–1.77)	< 0.001	1.42 (1.23–1.63)	< 0.001	1.41 (1.15–1.73)	0.001	1.19 (0.96–1.48)	0.107
	HVS_Q4	6217	429 (6.90)	1.42 (1.23–1.63)	< 0.001	1.34 (1.15–1.56)	< 0.001	1.20 (0.96–1.50)	0.110	0.98 (0.78–1.23)	0.846
	P for trend			< 0.001		0.001		0.042		0.829	
Total mortality	HVS_Q1	6778	396 (5.84)	ref		ref		ref		ref	
	HVS_Q2	7855	821 (10.45)	1.96 (1.74–2.21)	< 0.001	1.86 (1.64–2.11)	< 0.001	1.72 (1.45–2.06)	< 0.001	1.40 (1.17–1.68)	< 0.001
	HVS_Q3	6396	951 (14.87)	2.91 (2.58–3.27)	< 0.001	2.71 (2.39–3.06)	< 0.001	2.23 (1.87–2.66)	< 0.001	1.67 (1.39 – 2.00)	< 0.001
	HVS_Q4	6217	1,374 (22.10)	5.07 (4.53–5.67)	< 0.001	4.77 (4.23–5.38)	< 0.001	3.61 (3.04–4.29)	< 0.001	2.43 (2.03–2.90)	< 0.001
	P for trend			< 0.001		< 0.001		< 0.001		0.032	
Cardiac mortality	HVS_Q1	6778	85 (1.25)	ref		ref		ref		ref	
	HVS_Q2	7855	153 (1.95)	1.71 (1.31–2.23)	< 0.001	1.42 (1.08–1.89)	0.013	1.40 (1.00–1.97)	0.050	1.18 (0.84–1.67)	0.333
	HVS_Q3	6396	155 (2.42)	2.22 (1.70–2.89)	< 0.001	1.70 (1.28–2.26)	< 0.001	1.27 (0.89–1.81)	0.187	1.03 (0.72–1.48)	0.884
	HVS_Q4	6217	222 (3.57)	3.87 (3.01–4.96)	< 0.001	2.85 (2.16–3.76)	< 0.001	2.04 (1.44–2.88)	< 0.001	1.50 (1.06–2.14)	0.024
	P for trend			< 0.001		< 0.001		< 0.001		< 0.001	
Non-cardiac mortality	HVS_Q1	6778	311 (4.59)	ref		ref		ref		ref	
	HVS_Q2	7855	668 (8.50)	2.03 (1.78–2.32)	< 0.001	1.97 (1.71–2.26)	< 0.001	1.84 (1.49–2.26)	< 0.001	1.47 (1.20–1.82)	< 0.001
	HVS_Q3	6396	796 (12.45)	3.09 (2.71–3.53)	< 0.001	2.97 (2.59–3.40)	< 0.001	2.62 (2.14–3.22)	< 0.001	1.93 (1.56–2.37)	< 0.001
	HVS_Q4	6217	1,152 (18.53)	5.40 (4.76–6.12)	< 0.001	5.27 (4.61–6.03)	< 0.001	4.28 (3.550- 5.23)	< 0.001	2.80 (2.28–3.44)	< 0.001
	P for trend			< 0.001		< 0.001		< 0.001		< 0.001	

Model 1: no adjustment. Model 2: adjusted for age, gender, baseline BMI, HTN, COPD, CAD, PAOD, history of TIA/ischemic stroke, baseline FPG, baseline HbA1C, baseline eGFR; Model 3: adjusted for model2 plus echocardiography (LA size, LVEF, LV mass); Model 4: adjusted for model3 plus medications

Abbreviations as Table 1

**Fig. 2:**
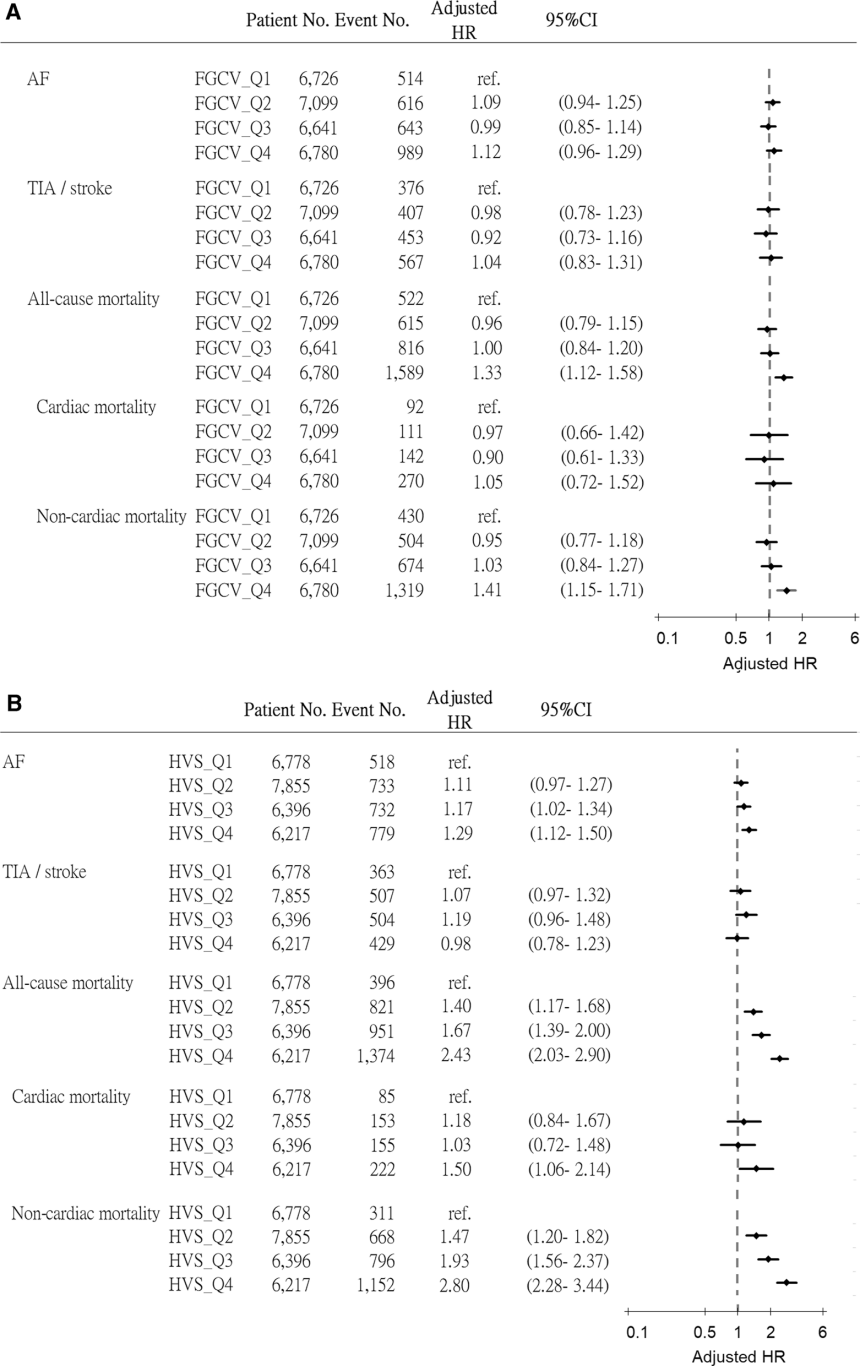
Forest plot of different outcomes in fully adjusted model stratified by quartiles of FGCV (**A**) or HVS (**B**)

Also shown in Table 2, by using the first quartile of HVS as reference, the HRs for AF remained significant in the third (1.17, 95% CI 1.02–1.34, p = 0.024) and fourth quartile (1.29, 95% CI 1.12–1.50, p < 0.001) in fully adjusted model. As for other outcomes, in fully adjusted model, in comparison with the first quartile, all three quartiles were significantly associated with total mortality and non-cardiac mortality but only the fourth quartile remained significant for cardiac mortality (1.50, 95% CI 1.06–2.14, p = 0.024). For TIA/ischemic stroke, all quartiles were nonsignificant in fully adjusted model. Forest plot of HRs in fully adjusted models were demonstrated in Fig. 2A (FGCV) and 2B (HVS). The numbers of FPG and HbA1c tests during follow-up were presented in Additional file 1).

**Fig. 3:**
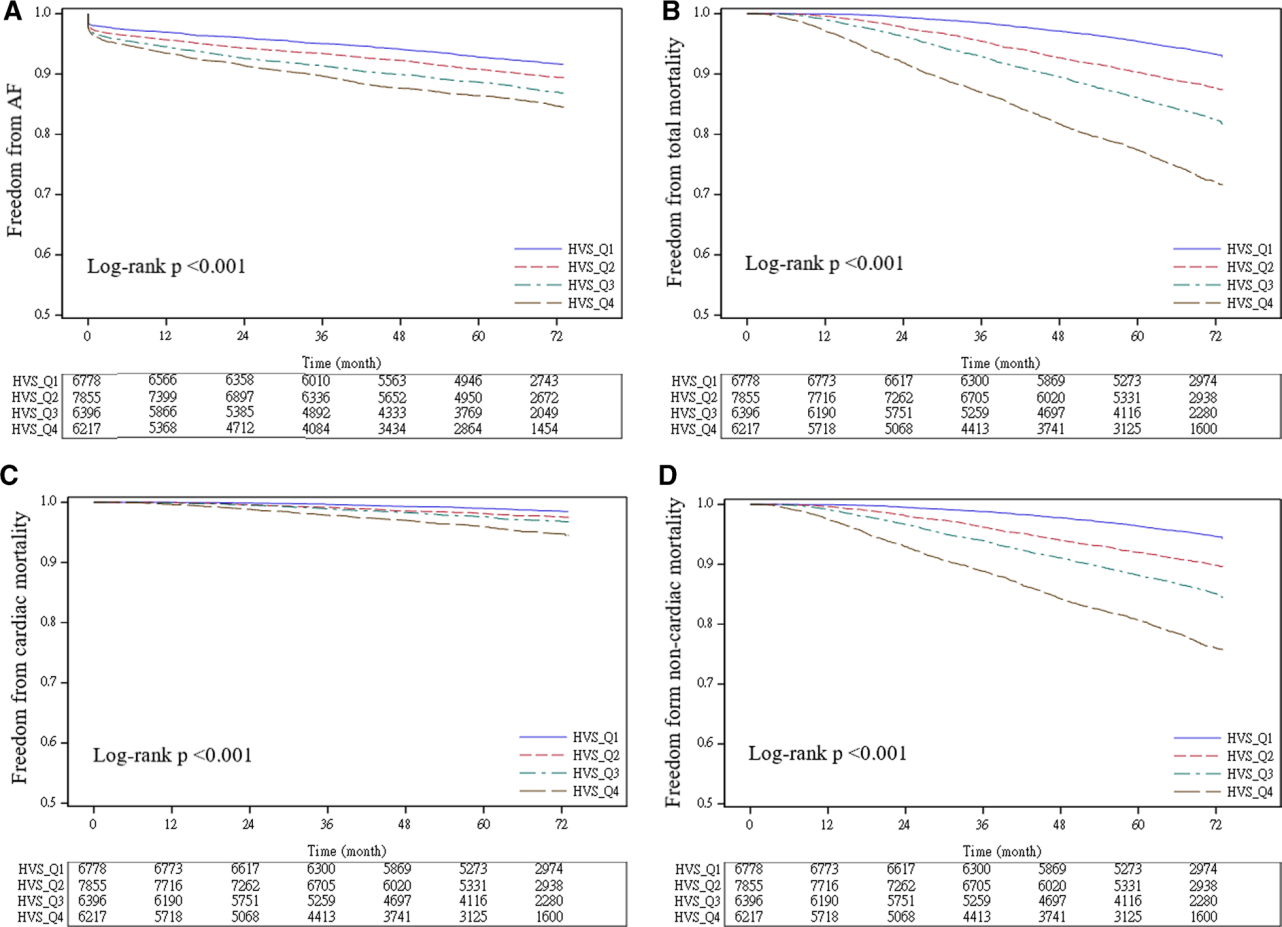
Kaplan–Meier analysis stratified by HVS for different outcomes: AF (**A**), total death (**B**), cardiac death (**C**) or non-cardiac death(**D**)

The results of Kaplan–Meier analysis were demonstrated in Fig. 3. As shown in Fig. 3, the probability of AF (3A), mortality (3B), non-cardiac mortality (3C) and cardiac mortality (3D) were significantly different across quartiles of HVS (All log-rank p < 0.001).

## Discussion

Our study showed that a greater GV is associated with a higher incidence of AF in patients with type 2 DM. In addition, a greater GV is independently associated with higher chances of cardiac and all-cause mortality. To our knowledge, this is the first cohort study to explore the association of long-term GV with the development of AF.

High GV has been proved to be associated with increased risk for cardiovascular events and poor prognosis [20, 21]. However, it’s impact on arrhythmia has been seldom studied. In a large Korea cohort of healthy population, the metabolic variability score composed of glucose level, blood pressure, total cholesterol level, and BMI showed a close association with the risk of AF, and the incidence was about 0.8 to 1.2 per 1000 person-year [22]. In our T2DM cohort, the overall incidence of AF was 21.31 per 1000 person-year, which was apparently much higher than the Asian healthy population without DM. The pathophysiology of AF development in DM has not been elaborately investigated. In a diabetic mice model, increased AF susceptibility was associated with reduced atrial conduction velocity, action potential duration prolongation, increased heterogeneity in repolarization, and increased interstitial atrial fibrosis [23]. In addition to blood sugar level, increased magnitude of GV may generate more reactive oxygen species than hyperglycemia alone. In diabetic rats, glucose fluctuations promote cardiac fibrosis by altering AKT signaling pathway and upregulate Txnip and NADPH oxidase expression which produce more reactive oxygen species levels, thereby increasing the incidence of AF [24, 25]. Other than direct effect, high GV may contribute to cardiac autonomic neuropathy which has a strong influence on cardiac arrhythmias [26].

Detrimental effects of glycemic fluctuations for AF are not only related to hyperglycemia but also hypoglycemia. Acute hypoglycemia is associated with the initiation and perpetuation of AF via mechanisms of increasing sympathetic activation, catecholamine surge and inflammation. Failure of intensive glycemic control to prevent new-onset AF in diabetic patients in the ACCORD trial hinted a potential harm that might come with hypoglycemia [27, 28]. Although the evidence for AF risk of hypoglycemia is circumstantial, a multitude of case reports of cardiac arrhythmias and electrocardiographic changes provoked by hypoglycemia had been reported [29].

The clinical meanings of the long-term and short-term GVs are different. Long-term GV is derived based on visit-to-visit measurements of HbA1c and FPG and is a marker of ambient hyperglycemia. In contrast, short-term GV represents episodes of either hyperglycemia or hypoglycemia within days [30]. In our study, we observed that HVS derived from HbA1c has better consistency and performance than FGCV derived from FPG in predicting future events. This implies that the impact of GV on AF development is a long-term cumulative process.

Long-term visit-to-visit HbA1c variability has been proved to be a strong predictor for both microvascular and macrovascular diseases and also for all-cause mortality [31, 32]. There are several ways to evaluate the HbA1c variability. One study showed that among the HbA1c variability parameters including mean of HbA1c, yearly mean HbA1c, HbA1c-SD, HbA1c-CV and HVS, HVS performed the best in predicting microvascular events [33]. One reason is that many of the HbA1c variability parameters are affected by the mean HbA1c value. For example, since the mean HbA1c is the denominator of the CV, intensive DM treatment may lower the mean value while increase this variability index [34]. HVS is defined as a percentage of HbA1c fluctuation events and is relatively insensitive to the change of the HbA1c absolute value and thus can independently provide accurate and stable GV information [19]. Our study also identified some patient characteristics that are subjective to high GV including male gender, high BMI, high baseline HbA1c and CKD. These factors could be an important reference for physicians who take care of patients with DM.

## Limitations

First, there might be surveillance bias because we did not screen for AF using a standardized, dedicated protocol at baseline or during follow-up period in this observative cohort. A recent study showed that the prevalence of asymptomatic, silent AF was 5–20% in diabetic subjects aged more than 65 years [35]. Since our patients were aged above 50 with a mean CHA2DS2-VASc score 2.56, the incidence of new-onset AF was possible to be underestimated. However, on the contrary, diabetic patients are more likely to have close follow-up, and thus they might have increased likelihood of being diagnosed with AF. The magnitude of these effects were difficult to be adjusted in this study. Second, we did not test all the reported GV parameters, such as average successive variability, average real variability of FPG, mean amplitude of glycemic excursion. We chose FGCV and HVS since they were commonly used, easily calculated parameters that could help physicians quickly determine the GV of their patients. Third, we excluded patients with severe end-organ damage including CHF and CKD to avoid complex AF confounders existing in these medical conditions. Whether the conclusion can be extrapolated to these conditions need further confirmation. Fourth, some known AF contributing factors, such as sleep apnea or sedentary behavior were not comprehensively evaluated in this study. Fifth, we excluded subjects who were not consistently followed at our out-patient clinics since the outcomes might be missing. This approach might cause selection bias but it could make sure that all the outcomes were accurately determined. Finally, this was a retrospective cohort study and the causal relationship might be less convincing.

## Conclusions

Our data demonstrated that high GV is independently associated with the development of new-onset AF in patients with T2DM. The benefit of maintaining stable glycemic levels to improve clinical outcomes warrants further studies (Additional file 1).

## Supplementary Information

**Additional file 1.** Supplementary data. **Figure S1.** Numbers of HbAc tests during follow-up. **Figure S2.** Numbers of fasting plasma glucose (FPG) tests during follow-up. **Table S1.** Median, mean, standard deviation (SD), minimum and maximum interval times (days) between HbA1c and FPG (fasting plasma glucose) tests.

## Data Availability

The datasets used in this study were only available in the National Taiwan University Hospital. The SAS programs (codes) involved for this study are available from the corresponding author on reasonable request.

## Abbreviations

AF: Atrial fibrillation
T2DM: Type 2 diabetes mellitus
GV: Glycemic variability
FGCV: Coefficients of variability of fasting glucose
HVS: HbA1c variability score
BMI: Body mass index
COPD: Chronic obstructive pulmonary disease
CAD: Coronary artery disease
PAOD: Peripheral arterial occlusive disease
FPG: Fasting plasma glucose
eGFR: Estimated glomerular filtration rate
LA: Left atrium
DT: Deceleration time
E/A: Early diastolic transmitral flow velocity/late diastolic transmitral flow velocity
E’: Early diastolic mitral annular velocity
LVEF: Left ventricular ejection fraction
LVIDd: Left ventricular internal diameter in diastole
LVIDs: Left ventricular internal diameter in systole
LV mass: Left ventricle mass
CCB: Calcium channel blocker
ACEI/ARB: Angiotensin converting enzyme inhibitor/angiotensin receptor blocker
SGLT-2 inhibitor: Sodium–glucose co-transporter-2 inhibitor
DPP4 inhibitor: Dipeptidyl peptidase 4 inhibitor
TZD: Thiazolidinediones
GLP-1 agonist: Glucagon like peptide-1 agonist
