# Supplementary material for: Higher long-term visit-to-visit glycemic variability predicts new-onset atrial fibrillation in patients with diabetes mellitus
Source: Cardiovasc Diabetol. 2021 Jul 23;20:148. doi: 10.1186/s12933-021-01341-3 (PMC8305511; doi:10.1186/s12933-021-01341-3)
Supplement: Supplementary file 1 — Additional file 1. Supplementary data. Figure S1. Numbers of HbAc tests during follow-up. Figure S2. Numbers of fasting plasma glucose (FPG) tests during follow-up. Table S1. Median, mean, standard deviation (SD), minimum and maximum interval times (days) between HbA1c and FPG (fasting plasma glucose) tests. [file 12933_2021_1341_MOESM1_ESM.docx]

**Additional file 1: Supplementary data. Figure S1. Numbers of HbAc tests during follow-up. Figure S2. Numbers of fasting plasma glucose (FPG) tests during follow-up. Table S1. Median, mean, standard deviation (SD), minimum and maximum interval times (days) between HbA1c and FPG (fasting plasma glucose) tests.**

Figure S1. Numbers of HbA1c tests during follow-up

Figure S2. Numbers of fasting plasma glucose tests (FPG) during follow-up

Table S1. Median, mean, standard deviation (SD), minimum and maximum interval times (days) between HbA1c and FPG (fasting plasma glucose) tests.

|  | Median | Mean | SD | Min | Max |
| --- | --- | --- | --- | --- | --- |
| HbA1C | 84 | 99.6 | 75.8 | 0 | 2073 |
| FPG | 84 | 93.4 | 75.3 | 0 | 2082 |
